# V̇O_2_peak estimation in people with overweight and obesity before and after a 14-week lifestyle intervention

**DOI:** 10.1038/s41366-025-01713-9

**Published:** 2025-01-20

**Authors:** Mikkel Thunestvedt Hansen, Karina Husted, Johanne Louise Modvig, Kristine Kjær Lange, Cecilie Moe Weinreich, Cathrine Tranberg, Tue Rømer, Arthur Ingersen, Flemming Dela, Jørn Wulff Helge

**Affiliations:** 1 Department of Biomedical Sciences, Faculty of Health and Medical Sciences, University of Copenhagen, Copenhagen, Denmark; 2 Xlab, Department of Biomedical Sciences, Faculty of Health and Medical Sciences, University of Copenhagen, Copenhagen, Denmark; 3 Laboratory of Sports and Nutrition Research, Riga Stradiņš University, Riga, Latvia

**Keywords:** Weight management, Lifestyle modification

## Abstract

**Purpose:**

This study aimed to investigate the validity and applicability of a non-exercise estimation of cardiorespiratory fitness using resting seismocardiography (SCG eV̇O_2_peak) in people with overweight and obesity before and after a 14-week lifestyle intervention.

**Methods:**

The study was carried out at a Folk high school that offers 14-week courses on lifestyle changes where participants live at the school and voluntarily participate in daily lectures and activities. Sixty-seven men and women with age and body mass index between 18 and 70 years and 25–50 kg·m^–2^ were tested at baseline, and 52 had a follow-up test after 14 weeks. Testing included the determination of anthropometric variables, an SCG eV̇O_2_peak at supine rest, and a gold standard V̇O_2_peak test on a cycle ergometer until voluntary exhaustion.

**Results:**

Agreement analysis for V̇O_2_peak at baseline (*n* = 67, SCG eV̇O_2_peak: 26.9 ± 1.9 ml·min^–1^·kg^–1^, V̇O_2_peak: 26.6 ± 1.6 ml·min^–1^·kg^–1^, mean ± 95% confidence interval) showed a bias of 0.3 ± 1.0 ml·min^–1^·kg^–1^ with 95% limits of agreement (LoA) ranging ± 9.8 ml·min^–1^·kg^–1^. A Pearson’s correlation of r = 0.78 (*p* < 0.0001) and a standard error of estimate (SEE) of 5.0 ml·min^–1^·kg^–1^ were found between methods. At follow-up (*n* = 52), body mass was reduced by 6.6 ± 1.4 kg (*p* < 0.0001). V̇O_2_peak increased by 3.3 ± 0.9 ml·min^–1^·kg^–1^ and 175 ± 78 ml·min^–1^ and SCG eV̇O_2_peak by 2.6 ± 0.8 ml·min^–1^·kg^–1^ and 93 ± 76 ml·min^–1^ (two-way ANOVA repeated measure: intervention *p* < 0.0001, method *p* = 0.939 and interaction *p* = 0.125, relative V̇O_2_peak). A Pearson’s correlation of r = 0.37 (*p* < 0.05) was found between changes in relative V̇O_2_peak but not for absolute V̇O_2_peak r = 0.10 (*p* = 0.402).

**Conclusions:**

The SCG method is accurate for estimating V̇O_2_peak and appropriate for detecting group changes in both relative and absolute V̇O_2_peak following a lifestyle intervention in people with overweight and obesity. Furthermore, the method can detect individual changes in V̇O_2_peak but not independently of body mass changes. Yet, the applicability is still limited by the relatively large variation in LoA and SEE.

## Introduction

Epidemiologic evidence strongly supports that cardiorespiratory fitness (i.e. V̇O_2_peak) is a powerful and independent predictor of morbidity and all-cause mortality beyond traditional risk factors [1–4]. Despite the well-established evidence and the 2016 scientific statement from the American Heart Association that V̇O_2_peak is a vital sign, V̇O_2_peak remains an underrated measure in both clinical and public health settings [5]. Directly measured pulmonary gas exchange rates during graded exercise testing until exhaustion (CPET) is the gold standard method for the determination of V̇O_2_peak [1]. The feasibility and use of the gold standard method is limited due to cost, time, exhausting exercise, and discomfort [5–7]. Non-exercise (N-Ex) V̇O_2_peak estimation equations constitute a feasible alternative with good applicability as a morbidity and all-cause mortality risk prediction tool [6–8]. However, N-Ex estimated V̇O_2_peak (eV̇O_2_peak) shows moderate accuracy when compared to V̇O_2_peak measured directly [9] and lacks accuracy in detection of V̇O_2_peak change over time [10]. This is even more pronounced when applied in subjects with a low V̇O_2_peak [9], a high BMI [11], or athletes [12], probably due to a weaker association between generally used determinants of V̇O2peak (age, sex, weight, and height), highlighting the need for better models within these populations. The N-Ex V̇O_2_peak estimation equations that include physical activity (PA) often perform better than those without, as PA is a major modifiable contributor to changes in V̇O_2_peak [1]. Physical activity is mostly self-reported as objectively measured PA limits the onsite feasibility of the eV̇O_2_peak, which is a limitation of the equations. Seismocardiography (SCG) measures precordial vibrations that originate from cardiac movement using an accelerometer, and the SCG wave-formed signal corresponds to the events in the cardiac cycle [13–15]. SCG thus provides an objective measure of cardiac function, which has been associated with exercise capacity [16, 17]. Time intervals and amplitudes in the SCG signal correlate with V̇O_2_peak [18] and a N-Ex V̇O_2_peak estimation equation using SCG at rest in combination with known anthropometric determinants of V̇O_2_peak has been developed (SCG eV̇O_2_peak) [18] and validated in healthy subjects [19, 20]. This study aimed to assess the accuracy and applicability of the established SCG V̇O_2_peak equation in a population with overweight and obesity before and after a 14-week lifestyle intervention by comparison between the obtained SCG eV̇O_2_peak value and the directly measured V̇O_2_peak value from a graded exercise test. Furthermore, the accuracy of the SCG eV̇O_2_peak was compared with two other established N-Ex V̇O_2_peak estimation equations.

## Materials and methods

### Study outline

This explorative study was part of another study investigating the proof of concept of a biological age model following a 14-week lifestyle intervention in people with overweight and obesity (unpublished). The study was carried out in the spring of 2022 (*n* = 46) and 2023 (*n* = 27) at “Ubberup Højskole” (UBH) in Denmark. UBH is a privately run Folk high school that offers a 14-week course on healthy living and lifestyle changes, where students live and voluntarily participate in daily lectures and activities at the school. The school typically enrolls around 70–80 students per course and most of the students have overweight or obesity. The study inclusion criteria were age above 18 years and a body mass index between 25 and 50 kg·m^–2^. The exclusion criteria were previous or current cardiovascular disease, pregnancy, and conditions preventing the performance of an exercise test to exhaustion. Participants received oral and written information about the study and the associated risks before written consent was obtained. This was an explorative study and not a confirmatory study of a well-defined hypothesis, therefore a sample size calculation was not conducted. The plan was to include as many participants as possible. The study adhered to the principles of the Helsinki Declaration and was approved by the Science Ethical Committee of the Greater Region of Copenhagen, Denmark (2022: H-19073643) and (2023: H-23012857). The intervention of the study is registered at ClinicalTrials.gov (NCT04279366).

### Lifestyle intervention

The course provides lectures on physical activity, health and nutrition, personal development, creative expression, and social studies and amounts to between 16 and 21 h of education per week. Furthermore, different physical activities, such as volleyball, swimming, cycling, walking, etc. are organized and provided daily. Food is also provided by the school with 4–6 daily meals and a particular emphasis is placed on healthy good-tasting food that is inspiring and stimulating to all the senses. It is recommended by the school that 50% of the plate should be vegetables, 25% starchy food, and 25% protein-containing foods. The students are provided with the opportunity to follow an individual diet, which guides them in portion size and structured eating times. It is the student’s own choice and responsibility to follow lectures, participate in activities, and/or follow the food guidance during the 14-week course.

### Overall experimental design

Testing was performed over three consecutive days in the first week and the last week of the 14-week course at UBH. Participants arrived in groups of five every 1.5 h from 07.00 to 11.30 following an overnight fast for measurement of anthropometry, body composition with bioimpedance (Tanita MC-780MA P, Tanita Corporation, Tokyo, Japan), blood pressure in supine position in triplicates separated by two minutes (Boso Medicus Control, BOSCH + SOHN GmbH u. Co. KG, Jungingen, Germany), estimation of V̇O_2_peak using SCG and completion of the International Physical Activity Questionnaire (IPAQ) short form for assessment of self-reported physical activity. Data from the body composition and blood pressure measurements will be presented as part of the biological age study (unpublished). The participants then arrived in the same groups of five on the same day for exercise testing occurring between 12.30 and 17.00 h.

### Estimation of V̇O_2_peak using Seismocardiography

The SCG V̇O_2_peak estimation procedures were performed as previously described [20]. Non-invasive cardiac vibrations on the sternum were recorded at supine rest with a small medical device (Seismofit®, VentriJect Aps, Hellerup, Denmark) containing a three-axis digital output accelerometer. The Seismofit device was placed with double adhesive tape on the sternum two centimeters proximal to the processus Xiphoideus. The Seismofit device is connected to a smartphone app with a cloud-based solution for SCG signal processing and algorithm-derived eV̇O_2_peak using before-entered age, sex, weight, and height of the participant in the app. The entire measurement lasts approximately three minutes (40-second SCG recording, 2 min data transferring and signal processing). The app then provides the estimated V̇O_2_peak score together with a resting heart rate, however, the recorded SCG signal is not available. The 4.6 version of the SCG V̇O_2_peak estimation model was used in this study (Schmidt et al., major revision in NPJ Cardiovascular Health 2024). Schmidt et al. will describe the development of this model version and it is presented in Table 2. When the influence of the SCG in the V̇O_2_peak model in the 4.6 database is assessed by comparing a regression model using only age, sex, weight, and height as variables with the model including SCG, the overall total explained variance increase from 0.65 to 0.76% in the model with SCG and the standard error of estimate (SEE), and mean absolute percentage error (MAPE) decrease from 6.1 ml·min^–1^·kg^–1^ to 5.0 ml·min^–1^·kg^–1^ and from 11.8% to 8.9%, respectively.

### Estimation of V̇O_2_peak using other non-exercise equations

Two other non-exercise V̇O_2_peak equations were chosen to compare the performance of the SCG V̇O_2_peak estimation model (Table 2). The equation by Schembre and Riebe [21] was used because this equation includes self-reported activity level obtained from IPAQ which was also obtained in the present study as a part of the biological age design. The equation by Myers et al. [22] was used as this is considered the best reference equation that uses the same demographic variables used in the SCG equation. In addition, the regression model without SCG and only demographics from the 4.6 database (No SCG eV̇O_2_peak) was applied to investigate the influence of SCG within the model in this population.

### V̇O_2_peak exercise testing

A graded exercise test protocol for determination of V̇O_2_peak was performed on a stationary cycle ergometer (Monark 839E, Monark Exercise AB, Vansbro, Sweden) with pulmonary gas exchange rates measured continuously using a mixing chamber and online equipment (Quark CPET, Cosmed, Rome, Italy). Pulmonary gas exchange measurements were obtained using 30-s running average with sampling every 10-s automatically by the software (Omnia, Cosmed, Rome, Italy). Two identical setups of cycle ergometers and online equipment were used simultaneously to keep up with the flow of participants. The applied exercise protocol was a 5-min warm-up at 30 W for women and 50 W for men, followed by increments of 20 W and 25 W every minute until voluntary exhaustion for women and men, respectively. Three experienced physiologists performed the testing and evaluation if the participant was at voluntary exhaustion. The participants were verbally familiarised with the test beforehand and encouraged throughout the entire test to exert their absolute best. The V̇O_2_peak was determined as the highest V̇O_2_ measured during 30 consecutive seconds. According to often-used test validity criteria, 69% of baseline and 65% of follow-up tests showed a VO_2_-plateau, defined as less than 2.1 ml·min^–1^·kg^–1^ with increasing workload. Only three baseline tests and three follow-up tests did not meet a VO_2_-plateau or at least two out of three secondary criteria (respiratory exchange ratio >1.1, within 10 bpm of age-predicted maximal heart rate, and 18≥ on the Borg 6–20 rate of perceived exertion scale) but these are included in the data analysis. The cycle ergometers and online equipment were calibrated according to the manufacturer’s instructions using a compressed gas mixture (5% CO_2_ and 16% O_2_) for the gas analyzers and a 3 L calibration syringe for the digital flowmeter in the mixing chamber setup, in the morning and after every other test. Participants were measured with the same equipment at baseline and at the follow-up test.

### Statistics

Data were normally distributed and presented as means ± 95% confidence intervals (CI) unless otherwise stated. The agreement and estimation error between measured and estimated V̇O_2_peak were analyzed using Bland-Altman plot (BA-plot) with 95% limits of agreement (LoA) [23], Standard Error of Estimate (SEE), and Mean Absolute Percentage Error (MAPE). Pearson product-moment correlation coefficient (Pearson’s r) was used to evaluate the relationship between V̇O_2_peak and eV̇O_2_peak. SEE was calculated using: ${SEE}=\surd \sum \frac{{\left(Y-{Y}^{{\prime} }\right)}^{2}}{n-2}$, with Y representing V̇O_2_peak and Y’ representing eV̇O_2_peak values. To evaluate the applicability of the eV̇O_2_peak equations, the agreement in classifying participants into V̇O_2_peak tertile groups was assessed at baseline and follow-up by Cohen’s κ coefficients and the 95% CI and interpreted as previously described [24]. The participants were divided into V̇O_2_peak tertiles based on age and sex using the Fitness Registry and the Importance of Exercise National Database (FRIEND) [25] and classified as having “lower” V̇O_2_peak if they were below the 33rd percentile, “mean” V̇O_2_peak if they were between the 33rd and 66th percentile, and “higher” V̇O_2_peak if they were above the 66th percentile. For participants completing the intervention, differences between baseline and follow-up were analyzed with a student’s paired t-test, except for V̇O_2_peak (absolute and relative) which was also analyzed across methods (eV̇O_2_peak and V̇O_2_peak) by two-way ANOVA repeated measures. Results from the two-way ANOVA are presented as mean difference and [95% CI of difference], with the intervention: follow-up (B)—baseline (A), method: measured (1)—estimated (2), interaction; (B1-A1)—(B2-A2). A significance level of α = 0.05 was applied. The performance of the eV̇O_2_peak equations in the detection of changes in V̇O_2_peak was based on Pearson’s r and BA-plot between changes. Absolute eV̇O_2_peak was found by multiplying the body mass of the participant to the estimate. Statistical analyses were performed, and figures were constructed in GraphPad Prism 10.1.1 (Software Inc., Boston, Massachusetts, USA) and Microsoft Excel (Microsoft Corporation, Redmond, Washington, USA).

## Results

The characteristics of the participants at baseline and the changes at follow-up are presented in Table 1.

**Table 1:** Descriptive characteristics of all participants at baseline and for the participants completing the follow-up test after the lifestyle intervention.

	All (*n* = 67)	Follow-up completers (*n* = 52)		
	Baseline	Baseline	Follow-up Change	Effect size
Women (%)	57	50	–	
**Anthropometry measurements**				
Age (yrs.)	40.7 ± 3.7	40.9 ± 4.1	0.2 ± 0.1*	0.15
Height (cm)	175 ± 2	176 ± 2	–	
Body mass (kg)	107.2 ± 5.6	109.3 ± 6.2	–6.6 ± 1.4***	0.63
Body mass index (kg·m^–2^)	34.6 ± 1.4	35.0 ± 1.6	–2.1 ± 0.4***	0.62
**Self-reported physical activity**				
Sitting time (MET min·week^–1^)	392 (343 to 447)^a^	383 (327 to 449)^b^	0.80 (0.67 to 0.96)^b^*	0.13
Walking (MET min·week^–1^)	274 (153 to 491)^a^	263 (135 to 513)^b^	1.84 (0.86 to 3.94)^b^	0.05
Moderate activity (MET min·week^–1^)	91 (42 to 197)^a^	85 (35 to 210)^b^	6.06 (2.28 to 16.10)^b^*	0.24
Vigorous activity (MET min·week^–1^)	42 (18 to 100)^a^	54 (20 to 143)^b^	16.22 (5.46 to 48.20)^b^***	0.36
**Non-Exercise estimation of** **V̇O**_**2**_**peak**				
SCG equation (ml·min^–1^·kg^–1^)	26.9 ± 1.9	27.3 ± 2.2	2.6 ± 0.8***	0.74
Myers et al. (ml·min^–1^·kg^–1^)	26.2 ± 1.9	26.5 ± 2.2	1.8 ± 0.4***	0.76
Schembre and Riebe (ml·min^–1^·kg^–1^)	40.0 ± 1.0^a^	40.5 ± 1.1^c^	2.8 ± 0.8***	0.65
**Measurement of exercise capacity**				
V̇O_2_peak (ml·min^–1^)	2796 ± 182	2899 ± 199	175 ± 78***	0.33
V̇O_2_peak (ml·min^–1^·kg^–1^)	26.6 ± 1.6	27.1 ± 1.9	3.3 ± 0.9***	0.74
Maximum ventilation (L·min^–1^)	116 ± 7	119 ± 8	4 ± 4***	0.08
Maximum load (watt)	208 ± 14	216 ± 14	19 ± 5***	0.53
Maximum heart rate (beats·min^–1^)	173 ± 5^d^	172 ± 6^e^	–3 ± 3***	0.06
Resting heart rate (beats·min^–1^)	62 ± 3	61 ± 2	–2 ± 2***	0.03
Maximum RPE	17.8 ± 0.3	17.9 ± 0.3	–0.1 ± 0.4	0.004
Maximum RER	1.19 ± 0.02	1.18 ± 0.02	0.02 ± 0.02***	0.10

Data are presented as mean ± 95% CI, except for self-reported physical activity which was not normally distributed and therefore is presented as geometric mean with lower and upper 95% CI of the geometric mean. *SCG* seismocardiography; *V̇O*_*2*_*peak* peak oxygen consumption; *RPE* rate of perceived exertion on the Borg 6-20 scale; *RER* respiratory exchange ratio. A twoway RM ANOVA with method (estimated and measured V̇O_2_peak) and intervention (baseline and follow-up) as the main effects were applied and the main effect of intervention are reported. A student's paired t-test was applied to assess the intervention's effect on other variables from the exercise test and the anthropometry measurements. A paired ratio t-test was performed on the self-reported physical activity. A significance level of α = 0.05 was applied. *p < 0.05, *** *p* < 0.0001. Effect size: partial eta square. a, *n* = 64, b, *n* = 47, c, *n* = 48, d, *n* = 59, e, *n* = 44.

### Agreement and estimation error of estimated V̇O_2_peak at baseline

**Fig. 1:**
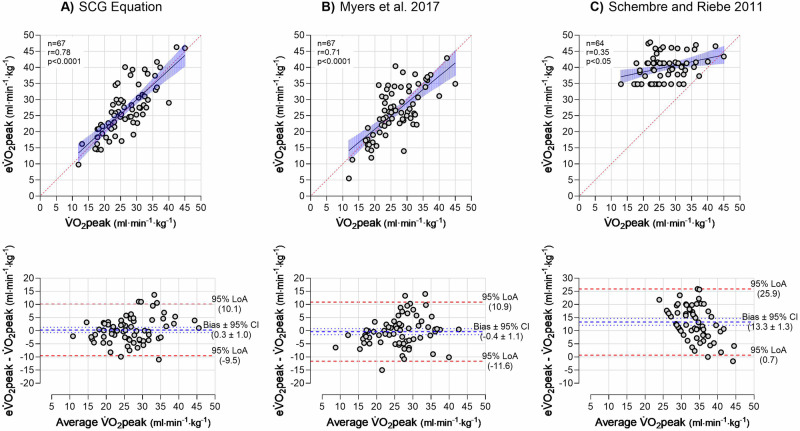
Scatter plots with linear regression (top) and Bland-Altman plots of the agreement (bottom) between estimated V̇O_2_peak and measured V̇O_2_peak. **A** V̇O_2_peak estimated with the SCG equation. **B** V̇O_2_peak estimated with the Myers et al. equation [22]. **C** V̇O_2_peak estimated with the Schembre and Riebe 2011 equation [20]. V̇O_2_peak peak oxygen consumption; eV̇O_2_peak estimated V̇O_2_peak; SCG seismocardiography; LoA limits of agreement; CI confidence interval. *n* = 67.

Compared with measured V̇O_2_peak a negligible bias with a considerable 95% LoA was found for V̇O_2_peak estimated with the SCG equation and the Myers et al. [22] equation (Fig. 1a, b, respectively), and both equations showed moderate estimation errors (Table 2). The eV̇O_2_peak using Schembre and Riebe [21] showed a significant bias with a considerable 95% LoA (Fig. 1c) and large estimation errors (Table 2).

**Table 2:** Accuracy of V̇O_2_peak estimations compared to directly measured V̇O_2_peak in people with overweight and obesity at baseline.

Algorithm	VO_2_peak estimation equation	Bias ± 95% CI	R^2^	SEE	MAPE
SCG 4.6	65.811 + (14.772·Sex [men = 1 & women = 0]) + (–0.229·Age) + (-0.300·Weight [kg]) + (0.092·Height [cm]) + (0.002·RR) + (4.671·Log_amp_AC_PeaktoPeak) + (-0.073·S2Morphology) + (0.197·S1FrequencySpec) + (-0.202·S2FrequencySpec) + (–70.894·amp_Ds) + (7.356·amp_range_s2_log_100) + (228.86·ampEs_40) + (–600.74·amp_s1_std_150)	0.3 ± 1.0	0.61	5.0	14.9
Myers et al. [22]	79.9–(0.39·age)–(13.7·sex [men=0 & women = 1])–(0.127·weight [lbs])	−0.4 ± 1.1	0.50	5.8	17.1
Schembre and Riebe 2011 [21]	47.749 – (6.493·sex [men = 1 & women = 2]) + (0.140·√vigorous activity [MET min·week^-1^])	13.3 ± 1.3***	0.13	15.0	57.9
No SCG	36.956 + (–0.302·Age) + (13.045·Sex [men = 1 & women = 0]) + (–0.458·Weight [kg]) + (0.257·Height [cm])	−0.3 ± 1.3	0.61	5.4	17.7

V̇O_2_peak is in ml·min^–1^·kg^–1^. 1 kg = 2.205 lbs. Vigorous activity is based on self-reported VPA with the International Physical Activity Questionnaire short form (IPAQ-S) and calculated into MET min·week^–1^ using the formula: VPA MET min·week^–1^ = 8.0 · VPA min · VPA days. ***SCG equation feature description***: RR, the average duration of a heartbeat at rest; Log_amp_AC peak to peak, log-transformed peak to peak amplitude in SCG diastolic complex; S2Morphology, morphology of the average SCG diastolic complex quantified using PCA; S1FrequencySpec, frequency of the average SCG systolic complex quantified using PCA; S2FrequencySpec, frequency of the average SCG diastolic complex quantified using PCA; amp_Ds, the amplitude of Ds point in the SCG signal; amp_range_s2_log_100, the amplitude of the diastolic complex after high pass filtering (100 Hz); ampEs_40, amplitude of Es point in SCG signal after high pass filtering (40 Hz), amp_s1_std_150, range of the amplitude of the systolic complex after high pass filtering (150 Hz). The No SCG equation is developed using 372 recordings from 221 subjects and validated in 138 recordings from 74 subjects from the 4.6 database, from which the SCG 4.6 is also developed. A significance level of α = 0.05 was applied. *** *p* < 0.001. *n* = 67 for the SCG equation (Schmidt et al. 2024, version 4.6), No SCG equation and Myers et al. [22], *n* = 64 for Schembre and Riebe 2011 [21].

*SCG* seismocardiography; eV̇O_2_peak estimated peak oxygen consumption; *SEE* standard error of estimation; *MAPE* mean absolute percentage error; *MET* metabolic equivalent of task; *PCA* principal component analysis; *VPA* vigorous physical activity.

### Changes in measured and estimated V̇O_2_peak at follow-up

**Fig. 2:**
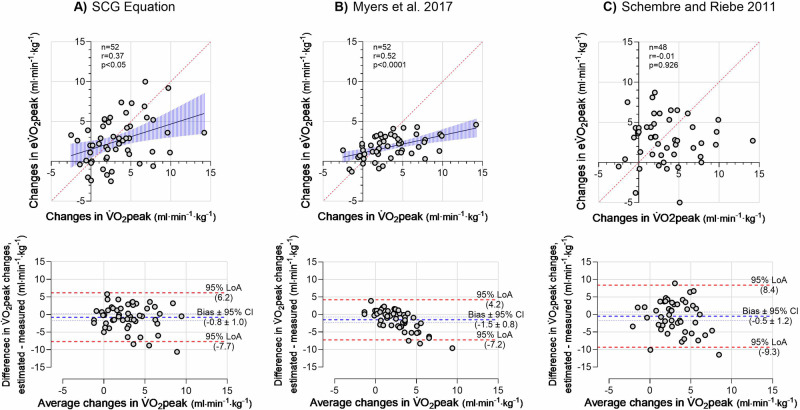
Scatter plots with linear regression (top) and Bland-Altman plots of the agreement (bottom) between changes in estimated V̇O_2_peak and measured V̇O_2_peak. **A** V̇O_2_peak estimated with the SCG equation. **B** V̇O_2_peak estimated with the Myers et al. 2017 equation [22]. **C** V̇O_2_peak estimated with the Schembre and Riebe 2011 equation [20]. V̇O_2_peak peak oxygen consumption; eV̇O_2_peak estimated V̇O_2_peak; SCG seismocardiography; LoA limits of agreement; CI confidence interval. *n* = 52.

Measured V̇O_2_peak increased by 12% and the SCG eV̇O_2_peak by 9% at follow-up with no difference between methods (Intervention: 2.9 ml·min^–1^·kg^–1^ [2.2 to 3.7], Method: 0.2 ml·min^–1^·kg^–1^ [–1.2 to 1.5], Interaction: 0.8 ml·min^–1^·kg^–1^ [–0.2 to 1.7]). An interaction effect was observed for the equation by Myers et al. (1.5 ml·min^–1^·kg^–1^ [0.7 to 2.3]) with a post hoc Šidák’s multiple comparisons test showing a 7% increase in eV̇O_2_peak (1.8 ml·min^-1^·kg^-1^ [1.1 to 2.5]) but a difference between methods at follow-up (2.1 ml·min^–1^·kg^–1^ [1.4–2.8]) and not at baseline (0.6 ml·min^–1^·kg^–1^ [–0.05 to 1.3]). The eV̇O_2_peak for Schembre and Riebe increased by 7% with a difference between methods and no interaction (Intervention: 3.0 ml·min^-1^·kg^-1^ [2.4 to 3.7], Method: –12.8 ml·min^–1^·kg^–1^ [–14.7 to –10.9], Interaction: 0.5 ml·min^–1^·kg^–1^ [–0.8 to 1.8]). Modest positive correlations and 95% LoA were observed between changes of V̇O_2_peak and eV̇O_2_peak values for the SCG equation and the equation by Myers et al. (Fig. 2A, B), whereas no correlation and large 95% LoA were observed for the equation by Schembre and Riebe (Fig. 2C). Body mass was reduced by 6% at follow-up (–6.6 kg [–8.0 to –5.2]). Absolute V̇O_2_peak increased by 175 ml·min^–1^ [97 to 253] and eV̇O_2_peak increased by 93 ml·min^–1^ [17 to 169] for the SCG equation and was unchanged for the equations by Myers et al. (18 ml·min^–1^ [–5 to 42]) and Schembre and Riebe (23 ml·min^–1^ [–91 to 137]). There was no difference between absolute V̇O_2_peak and SCG eV̇O_2_peak (Intervention: 134 ml·min^–1^ [76–192], Method: 27 ml·min^–1^ [–125 to 179], Interaction: 82 ml·min^–1^ [–25 to 189]). There was no correlation between changes in absolute V̇O_2_peak and SCG eV̇O_2_peak (r = 0.10, *p* = 0.402).

### Performance of the eV̇O_2_peak Equations

At baseline, 81% of all participants were categorized into the correct V̇O_2_peak tertile group with SCG eV̇O_2_peak showing a substantial agreement between methods (Table 3). For the equation by Myers et al., there was a moderate agreement with 72% correctly categorized, and for Schembre and Riebe there was no agreement with 15% correctly categorized (Table 3). The agreement for correct V̇O_2_peak categorizations at follow-up was moderate (73%), fair (58%), and slight (25%) for SCG, Myers et al., and Schembre and Riebe, respectively (Table 3). Nine participants changed the V̇O_2_peak tertile group, six from “lower” to “mean” and three from “mean” to “higher”. The SCG V̇O_2_peak estimation equation correctly estimated seven of these nine participants with group changes (five “lower” to “mean” and two “mean” to “higher”), and Myers et al. correctly estimated three from “lower” to “mean” and Schembre and Riebe one from “mean” to “higher”.

**Table 3:** Classification of participants into three V̇O_2_peak categories according to measured V̇O_2_peak and estimated V̇O_2_peak at baseline and follow-up.

		SCG eV̇O_2_peak			Myers et al. eV̇O_2_peak			Schembre and Riebe eV̇O_2_peak		
		Lower	Average	Higher	Lower	Average	Higher	Lower	Average	Higher
Baseline V̇O_2_peak	Lower	**37** (93)	3 (8)	0 (0)	**36** (90)	4 (10)	0 (0)	**0** (0)	29 (74)	10 (26)
	Average	6 (29)	**15** (71)	0 (0)	9 (43)	**12** (57)	0 (0)	0 (0)	**5** (26)	14 (74)
	Higher	0 (0)	4 (67)	**2** (33)	1 (17)	5 (83)	**0** (0)	0 (0)	1 (17)	**5** (83)
	Cohen’s Kappa	κ = 0.62 [0.45 to 0.80]			κ = 0.42 [0.23 to 0.61]			κ = − 0.06 [−0.16 to 0.03]		
Intervention baseline V̇O_2_peak	Lower	**29** (91)	3 (9)	0 (0)	**28** (88)	4 (13)	0 (0)	**0** (0)	24 (80)	6 (20)
	Average	5 (33)	**10** (67)	0 (0)	7 (47)	**8** (53)	0 (0)	0 (0)	**4** (31)	9 (69)
	Higher	0 (0)	3 (60)	**2** (40)	1 (20)	4 (80)	**0** (0)	0 (0)	1 (20)	**4** (80)
	Cohen’s Kappa	κ = 0.58 [0.37 to 0.79]			κ = 0.37 [0.15 to 0.59]			κ = − 0.05 [−0.16 to 0.07]		
Intervention follow-up V̇O_2_peak	Lower	**21** (81)	5 (19)	0 (0)	**21** (81)	5 (19)	0 (0)	**0** (0)	12 (50)	12 (50)
	Average	4 (22)	**11** (61)	3 (17)	9 (50)	**9** (50)	0 (0)	0 (0)	**6** (35)	11 (65)
	Higher	0 (0)	2 (25)	**6** (75)	1 (13)	7 (88)	**0** (0)	0 (0)	0 (0)	**7** (100)
	Cohen’s Kappa	κ = 0.56 [0.37 to 0.76]			κ = 0.25 [0.04 to 0.45]			κ = 0.06 [−0.05 to 0.17]		

Data are presented as counts and (percentages). κ = Cohen’s Kappa coefficient and [95% CI]. The reference standards from the Fitness Registry and the Importance of Exercise National Database (FRIEND) were used to divide participants based on age and sex into the V̇O_2_peak tertile group [24]. The three V̇O_2_peak categories were determined as “lower” if the V̇O_2_peak was below the 33rd percentile, “average” if V̇O_2_peak was between the 33rd and 66th percentile, and “higher” if V̇O_2_peak was above the 66th percentile. V̇O_2_peak is in ml·min^–1^·kg^–1^.

*SCG* seismocardiography; *V̇O*_*2*_*peak* peak oxygen consumption; *eV̇O*_*2*_*peak* estimated V̇O_2_peak.

## Discussion

This study provides novel insight into the accuracy and applicability of eV̇O_2_peak at rest using the SCG method before and after a lifestyle intervention in a healthy population with overweight and obesity. The SCG eV̇O_2_peak is accurate in determining V̇O_2_peak on a group level, although a considerable 95% LoA is present, and it can detect V̇O_2_peak changes following a lifestyle intervention. Compared with the eV̇O_2_peak by the equations by Myers et al. and Schembre and Riebe the authors consider the SCG V̇O_2_peak estimation equation to be superior.

### Agreement in V̇O_2_peak estimation

What is a good and acceptable method agreement for V̇O_2_peak determination? A degree of error of more than one metabolic equivalent (1 MET ~ 3.5 ml·min^–1^·kg^–1^) could impact risk classification and treatment, as one MET improvement reduces mortality [1, 26]. However, obtaining an estimation error within 3.5 ml·min^-1^·kg^-1^ is a tough criterion when considering the intra-individual variation within the gold standard measure, typically is reported around 5% [27]. A recent study has cross-validated 28 N-Ex V̇O_2_peak estimation equations within a single cohort (*n* = 2529) to determine which equations were the most accurate [9]. All equations showed significant correlations with R^2^ ranging between 0.25 to 0.70 and SEE between 4.1 and 6.2 ml·min^–1^·kg^–1^ [9]. Interestingly, eV̇O_2_peak was significantly different from V̇O_2_peak in 27 out of the 28 equations with LoA ranging between ±10.4 and ±15.4 ml·min^–1^·kg^–1^ [9]. This certainly addresses the limitations when using V̇O_2_peak estimation equations. In addition, an unfortunate and further limitation of V̇O_2_peak estimation equations is a weaker agreement among individuals with lower V̇O_2_peak, where the associated risk of mortality is higher. This is supported by the finding that only 47% of the participants were correctly classified into the lowest tertile on average for the 28 equations [9]. In comparison to both the performance in the abovementioned cross-validation and the application of two of these equations within this study, the agreement of the SCG eV̇O_2_peak is better based on an R^2^ of 0.61, SEE of 5.0 ml·min^-1^·kg^-1^, no difference between methods, LoA ranging 9.8 ml·min^–1^·kg^–1^, and 81% of the participants correctly classified into V̇O_2_peak groups with a Cohen’s κ of 0.62. V̇O_2_peak estimation equations that include physical activity are often performing better, due to physical activity being a major modifiable contributor to changes in V̇O_2_peak [1]. However, the equation by Schembre and Riebe largely overestimated V̇O_2_peak in the present study, clearly emphasizing the limitation of self-reported physical activity [28], which is perhaps also more pronounced in individuals with body mass index >25 kg·m^–2^ [29]. Objectively measured physical activity is preferred compared to self-reported, but it also limits the onsite applicability of eV̇O_2_peak. The SCG provides a measure of resting intrinsic cardiac performance with onsite applicability. Compared with the Myers et al. equation, the SCG equation only showed a slightly better agreement and correct V̇O_2_peak group categorization, which was attributed to better performance in individuals with higher V̇O_2_peak values. This could indicate that the SCG can capture some of the variation in V̇O_2_peak that is not explained by age, sex, and weight. Together, these results favor the application of the SCG eV̇O_2_peak for the determination of V̇O_2_peak in a healthy population with overweight and obesity when the gold standard method is not feasible.

### Detection of change in V̇O_2_peak

V̇O_2_peak equations that estimate group changes accurately can be useful in a research setting, however, applicability within the clinical setting requires accurate estimation of the individual changes [9]. V̇O_2_peak estimation equations without physical activity in general have a poor ability to detect changes over a “short” time, due to age and anthropometry normally remaining relatively unchanged. This is not surprising given the importance of physical activity for changes in V̇O_2_peak [1], which, widely accepted, is due to central adaptation through a change in cardiac output (i.e. change in stroke volume contributed by a change in the diastolic filling, Frank-Starling Law). In this study, an average reduction in body mass was present, which together with age (seven participants chronologically aged one year) allowed the equation by Myers et al. to detect a change in eV̇O_2_peak. However, when eV̇O_2_peak was expressed as an absolute eV̇O_2_peak, no difference was observed with the Myers et al. equation, whereas absolute eV̇O_2_peak still increased with the SCG equation, even though body mass is also a factor within that equation. This variation can either be attributed to the added SCG within the equation or because the body mass does not have the same weighting within the equation. It is, therefore, difficult to explain what is driving this observed discrepancy. The fact that there was no correlation between changes in absolute SCG eV̇O_2_peak and V̇O_2_peak indicates that the SCG method is not sensitive enough to detect actual changes in V̇O_2_peak independently of body weight following the intervention and that the demographic components in the model still contribute to a great extent. For the Schembre and Riebe equation, body mass is not integrated and the reported vigorous physical activity, therefore, drives the improved eV̇O_2_peak. However, there was no correlation in V̇O_2_peak changes, no agreement in classifying the correct V̇O_2_peak category, and no improvement when expressed in absolute eV̇O_2_peak thereby indicating a poor agreement in detecting the individual changes and group changes independently of a body mass change. The SCG V̇O_2_peak equation performed better in detecting the change between the “lower”, “mean”, and “higher” V̇O_2_peak categories than the other equations, emphasizing better agreement with this method.

## Perspectives

The present findings support the use of the SCG eV̇O_2_peak when conducting both cross-sectional and longitudinal studies in large cohorts with body mass index >25 kg·m^-2^, where the gold standard directly measured V̇O_2_peak is not possible. The SCG methodology would be interesting to evaluate in a population with heart disease, where the cardiac function is limiting V̇O_2_peak, and thus other N-Ex V̇O_2_peak estimation equations are not appropriate.

## Limitations

The explorative nature of the study has limitations, however, it provides a foundation for more controlled and conclusive studies in the future. A limitation is the lack of control for hydration status and subcutaneous tissue thickness, which might be interfering factors in the assessment of V̇O_2_peak with the methods. The lack of SCG information when performing the estimation is a profound limitation and limits the possibility of assessing the actual influence of the SCG in the V̇O_2_peak equation in this cohort. The relatively large number of missed participants at follow-up (22%) is a limitation. Another limitation is the use of the equation by Schembre and Reibe, but there are no other N-Ex V̇O_2_peak estimation equations available that use IPAQ estimated PA and have a good overall agreement. Finally, the researchers were not blinded by the SCG eV̇O_2_peak and this could potentially influence how the V̇O_2_peak test was conducted. However, the criteria of the V̇O_2_peak test were assessed, and an equal distribution of data around the line of identity would suggest that a fair V̇O_2_peak assessment has been performed.

## Conclusion

The SCG equation is accurate for the estimation of V̇O_2_peak in a healthy population with overweight and obesity, and the method is appropriate for detecting group changes in both relative and absolute V̇O_2_peak following a 14-week lifestyle intervention. Furthermore, the method is able to detect individual changes in V̇O_2_peak but not independently of body mass changes. Yet, the applicability is still limited by the relatively large variation in LoA and SEE.

## Data Availability

Data used in the current study are available upon reasonable request.
